# Mutual Skill Learning and Adaptability to Others via Haptic Interaction

**DOI:** 10.3389/fnbot.2021.760132

**Published:** 2021-12-02

**Authors:** Ozge Ozlem Saracbasi, William Harwin, Toshiyuki Kondo, Yoshikatsu Hayashi

**Affiliations:** ^1^Biomedical Sciences and Biomedical Engineering, School of Biological Sciences, University of Reading, Reading, United Kingdom; ^2^Department of Computer and Information Sciences, Graduate School of Engineering, Tokyo University of Agriculture and Technology, Tokyo, Japan

**Keywords:** physical human-robot interaction, human-human interaction, collaborative learning, motor learning, skill learning, adaptability

## Abstract

When learning a new skill through an unknown environment, should we practice alone, or together with another beginner, or learn from the expert? It is normally helpful to have an expert guiding through unknown environmental dynamics. The guidance from the expert is fundamentally based on mutual interactions. From the perspective of the beginner, one needs to face dual unknown dynamics of the environment and motor coordination of the expert. In a cooperative visuo-haptic motor task, we asked novice participants to bring a virtual mass onto the specified target location under an unknown external force field. The task was completed by an individual or with an expert or another novice. In addition to evaluation of the motor performance, we evaluated the adaptability of the novice participants to a new partner while attempting to achieve a common goal together. The experiment was set in five phases; baseline for skill transfer and adaptability, learning and evaluation for adaptability and skill transfer respectively. The performance of the participants was characterized by using the time to target, effort index, and length of the trajectory. Experimental results suggested that (1) peer-to-peer interactions among paired beginners enhanced the motor learning most, (2) individuals practicing on their own (learning as a single) showed better motor learning than practicing under the expert's guidance, and (3) regarding the adaptability, peer-to-peer interactions induced higher adaptability to a new partner than the novice-to-expert interactions while attempting to achieve a common goal together. Thus, we conclude that the peer-to-peer interactions under a collaborative task can realize the best motor learning of the motor skills through the new environmental dynamics, and adaptability to others in order to achieve a goal together. We suggest that the peer-to-peer learning can induce both adaptability to others and learning of motor skills through the unknown environmental dynamics under mutual interactions. On the other hand, during the peer-to-peer interactions, the novice can learn how to coordinate motion with his/her partner (even though one is a new partner), and thus, is able to learn the motor skills through new environmental dynamics.

## 1. Introduction

As social beings, humans need to learn a variety of motor skills to perform everyday tasks. Skilled motor behavior is necessary for many human activities, such as daily life activities (e.g., driving), sport activities (e.g., basketball), art performances (e.g., playing musical instruments), and occupations (e.g., surgery). Auditory (Konvalinka et al., 2010; Wolf et al., 2018), visual (Newman-Norlund et al., 2008) or haptic feedback (van der Wel et al., 2011; Madan et al., 2014; Takagi et al., 2017; Özen et al., 2020) is important to coordinate actions and learn new skill sets in a cooperative task, such as ensemble music performance or dancing.

From birth, humans learn how to use motor skills and control their movements. In motor learning tasks, transferring a skill from an expert (a teacher or a coach) to a novice (a learner or a player) plays an important role. Learning and teaching (Bremmer and Nijs, 2020), which are mutually complementary terms associated with an interactive experience between the expert and the novice, appear in physical activities, such as dance as well as in rehabilitation. For example, in teaching dance, an expert may teach a novice how to dance by using the haptic interaction associated with moving in synchrony or by guiding desired movements. Physical therapists guide their patients *via* haptic interaction to help the person learn or relearn specific movements (Sawers and Ting, 2014). In rehabilitation, in addition to physical therapy provided by therapists, the recent technological advancements paved the way for the usage of robotic systems in helping humans to improve their motor skills and motor recovery and robot-assisted therapy in stroke rehabilitation (Wei et al., 2005).

More generally, in recent years, there has been a growing interest to create robots having the ability to interact with humans in a more natural manner (Nasr et al., 2020). It is important to create robotic systems which can provide more natural human-robot interactions. Thus, first, understanding the nature of human-human interaction is an important step, i.e., understanding in such a way that how a coach, athletic trainer, teacher, or physical/occupational therapist facilitate the learning process (Ganesh et al., 2014; Sawers and Ting, 2014; Mireles et al., 2017; Takagi et al., 2017). The previous studies (Ganesh et al., 2014; Beckers et al., 2018) found that a paired performance is more advantageous than an individual performance in motor learning, which has been proved by connecting two participants to each other *via* virtual spring while tracking the virtual target by controlling a haptic interface.

Here, a haptic interface (Hernantes et al., 2012) is a device that includes a robotic mechanism along with sensors to determine position of humans in the virtual environment and actuators to apply forces to the operator and is used to manipulate an object within a virtual environment. The usage of robotic haptic interfaces generating force-field paved the way for understanding the human mechanism while learning skills through dyadic haptic interaction. In the case of dyadic interaction, humans can face a dual instability arising both from the environment and the interaction with a partner (De Santis et al., 2014; Mireles et al., 2017). As shown in the cases above, it is normally helpful for novice participants to learn the motor skills through the unknown environmental dynamics with an expert guiding through unknown environmental dynamics. However, as the guidance from the expert is fundamentally based on mutual interactions, from the perspective of the beginner, one needs to simultaneously face dual unknown dynamics of the environment and motor coordination of the expert. When the beginner learns a task with a partner, one needs to learn how to coordinate the body motion, predicting the next motion of the partner. This inevitably involves the process of adaptation to the partner. Here, as opposed to the normal assumption where the guidance from the expert is always helpful, we hypothesize that learning with another beginner would enhance motor learning as a result of peer-to-peer learning, adapting to the other's dynamics, and exploring the unknown environmental dynamics together. Thus, the fundamental question is, when learning a new motor skill, whether we should practice alone, or learn from the expert, or learn together with another beginner. To date, several studies (Masumoto and Inui, 2013; Ganesh et al., 2014; Mireles et al., 2017; Kostrubiec et al., 2018) investigated skill learning by comparing paired performance and individual performance, and found that the paired one showed better motor performance than the individual one. The studies indicating the importance of dyadic interaction in skill learning lead us to consider the effect of the interacting partner on skill learning. The recent study (Mireles et al., 2017) employing novice-to-novice and novice-to-expert interactions suggested that in cooperative tasks the best performances were induced during the training with an expert, but the novices trained with an expert were not able to perform the task well when the expert is removed. That is to say, the study (Mireles et al., 2017) highlighted the importance of exploration of the environmental dynamics in the cooperative task for skill learning. The research to date (Shadmehr and Mussa-Ivaldi, 1994; Krakauer et al., 1999; Sakamoto and Kondo, 2015) focused on skill learning mostly through adaptation to virtual force fields or visuomotor transformations in reaching tasks. However, how a human learns to adapt to the dynamics of a partner during the novice-to-novice interaction remains still unclear.

Skill learning is the result of the interactions between the learner (novice) and the learning environment (Bremmer and Nijs, 2020), so in the individual performance, the improvement depends on the adaptation to the environmental dynamics, whereas in the dyadic interaction, it depends on not only adaptation to the environmental dynamics but also dynamics of partner (Magill and Anderson, 2010; Jundt et al., 2015). For example, in paired skating, to perform common trajectories on the ice ground, an ice skater is trained to adapt to the environmental factors and understand the actions of the partner.

In the previous literature, the confusion originates from the fact that skill transfer and mutual interactions were studied independently. It means that much uncertainty still remains about the nature of motor skill learning under the unknown environment where it inevitably involves mutual interactions. Thus, we aim to study the relationship between skill learning and adaptability to others, and thus, seek for the conditions which can induce the best motor learning. To this end, a cooperative task using a backdrivable haptic device will be employed for participants to achieve a common goal under unknown environmental dynamics. The ability to adapt to the motor coordination of other participants (adaptability to others) should be a key to exploring the unknown environmental dynamics, and thus, learning the motor skill to achieve a goal under the unknown dynamics.

We hypothesized that there should be a positive correlation between skill learning and adaptability, which means that the participants in Novice-to-Novice groups can adapt to each other by exploring the unknown environmental dynamics together, and motor skill learning can happen only when there is adaptation.

**Figure 1:**
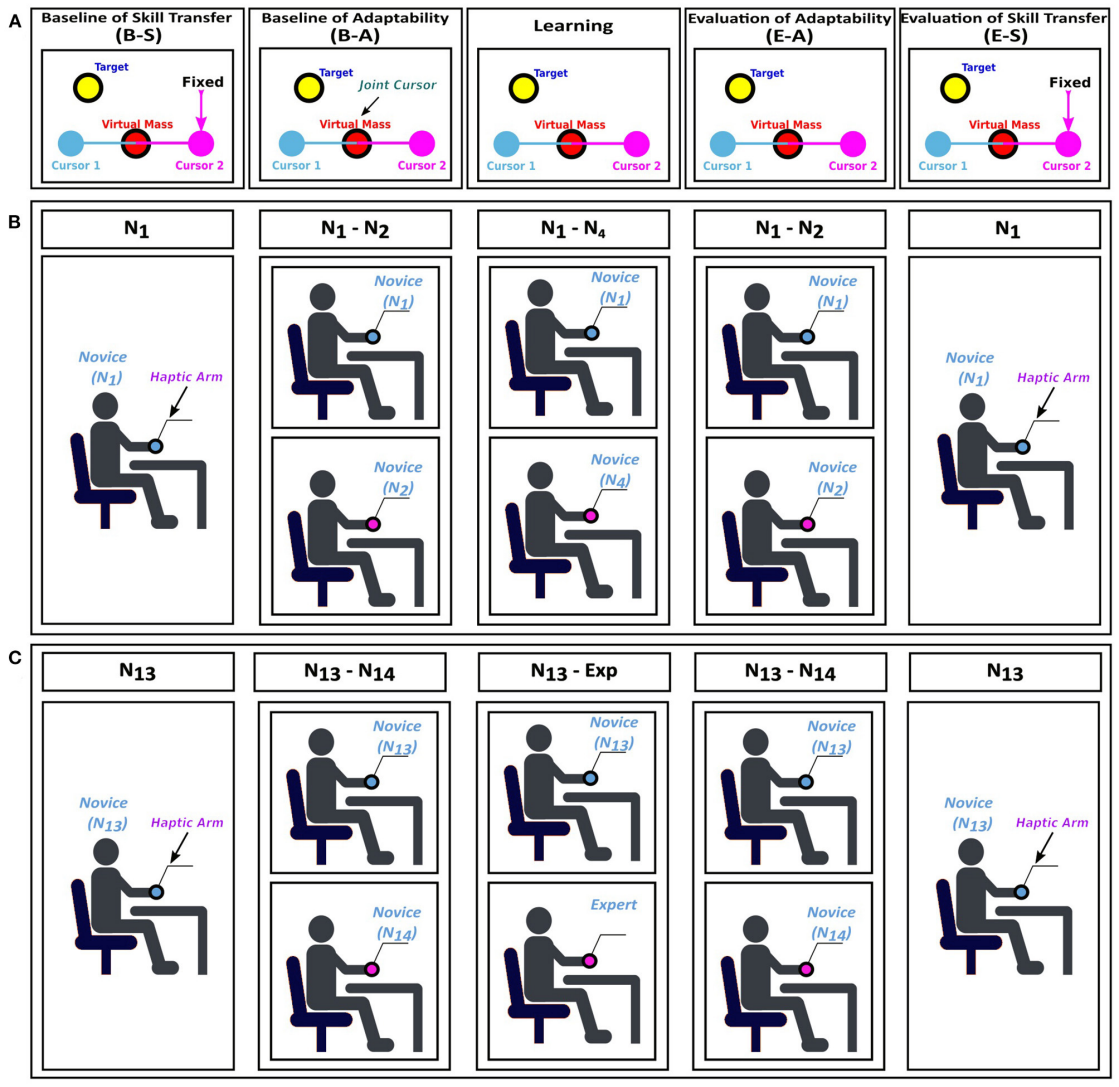
Experimental protocol. **(A)** The structure of the virtual tool and the experimental phases for the Novice-Novice (*N-N*) and Novice-Expert (*N-E*) groups: Baseline of Skill Transfer (B-S), Baseline of Adaptability (B-A), Learning, Evaluation of Adaptability (E-A), and Evaluation of Skill Transfer (E-S). **(B)** *N-N* group; the Learning phase was performed with a new novice participant (here, *N1* and *N4*). **(C)** *N-E* group; the Learning phase was performed with an expert (here, *N13* and *Exp*).

**Figure 2:**
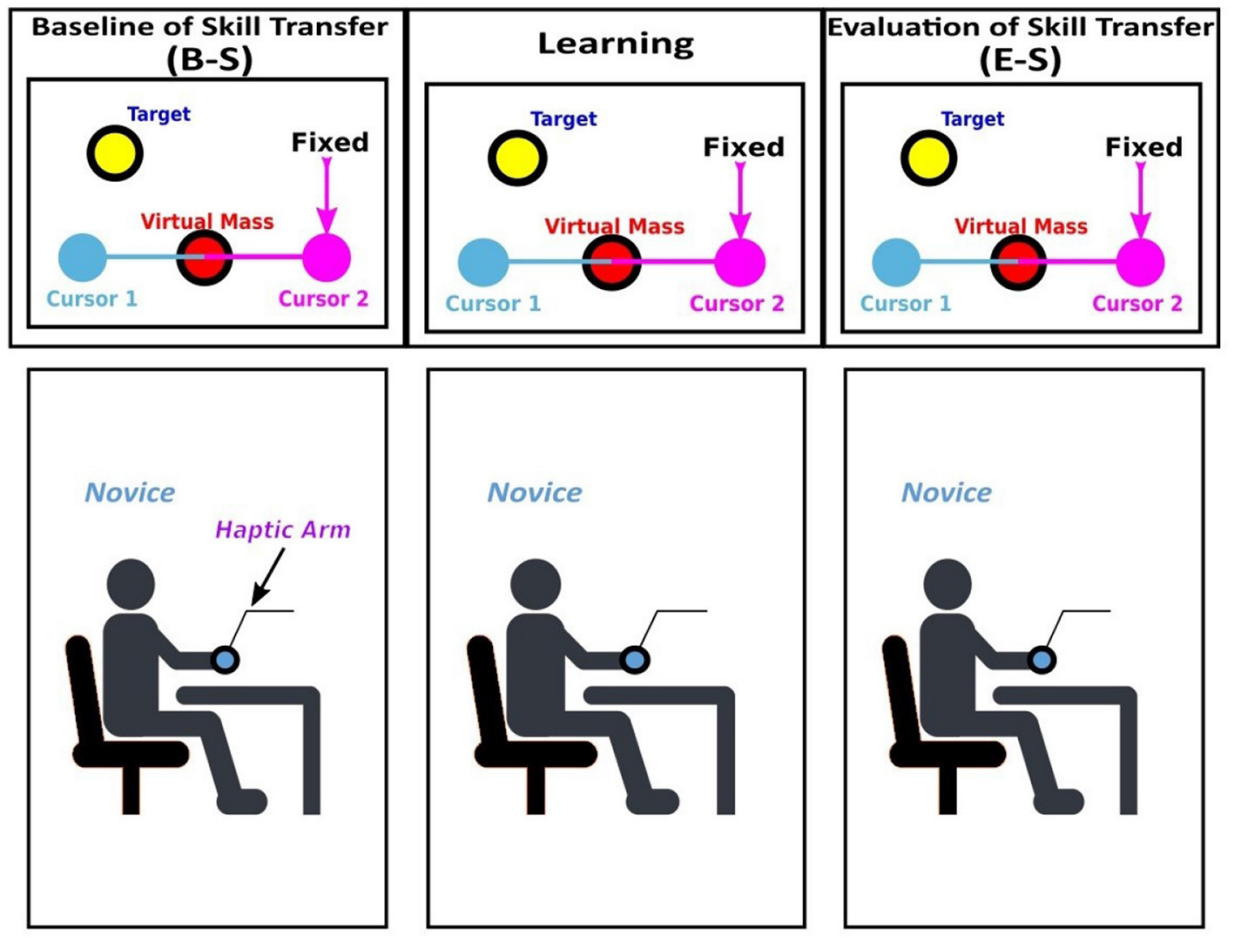
Experimental protocol; the structure of the virtual tool and the experimental phases for the *Alone* group. Baseline of Skill Transfer (B-S), Learning and Evaluation of Skill Transfer (E-S). In each phase, the position of cursor 2 (purple circle) was fixed at a certain location and the cursor 1 (blue circle) which was correlated with motion of the end-effector of the haptic arm. The novice participant was asked to move a virtual mass (red circle) to the target position (yellow circle) as a single by using the haptic arm.

In the study, we adopted the widely used paradigm (for example, see Mireles et al., 2017), first to investigate the effect of practicing alone or training with the expert or novice partner on motor performance. Second, we studied “adaptability to others,” introducing Evaluation of Adaptability (see Table 1) as a new experimental protocol of the participant experiments. In this paradigm, we asked the participants to guide a virtual mass to bring it to a specified target under an external force field as an individual or with their preassigned partner (expert or novice). The novice participants were engaged to learn the motor skills, manipulating the haptic device under the unknown force field. Using the detected time interval, force and trajectories during the task, we evaluated the motor learning of the novice group trained with the expert or another novice as well as the degree of adaptability. Details of the experiment are given in Figures 1, 2 and the section 2.

**Table 1:** Experimental protocol.

**(A) Novice-Novice (N-N) and Novice-Expert (N-E) groups**
	**Baseline of**	**Baseline of**		**Evaluation of**	**Evaluation of**
	**skill transfer**	**adaptability**	**Learning**	**adaptability**	**skill transfer**
	**B-S**	**B-A**		**E-A**	**E-S**
	**(1-FS, 2-TS)**	**(2-FS, 1-TS)**	**(2-FS, 30-TS)**	**(2-FS, 6-TS, 2-WS)**	**(4-TS)**
	*N*_1_ |*N*_2_	*N*_1_ - *N*_2_	*N*_1_ - *N*_4_	*N*_1_ - *N*_2_	*N*_1_ |*N*_2_
(N-N)	*N*_3_ |*N*_4_	*N*_3_ - *N*_4_	*N*_2_ - *N*_3_	*N*_3_ - *N*_4_	*N*_3_ |*N*_4_
(*n* = 12)	⋮	⋮	⋮	⋮	⋮
	*N*_13_ |*N*_14_	*N*_13_ - *N*_14_	*N*_13_ - *Exp*, *Exp*- *N*_14_	*N*_13_ - *N*_14_	*N*_13_ |*N*_14_
(N-E)	*N*_15_ |*N*_16_	*N*_15_ - *N*_16_	*N*_15_ - *Exp*, *Exp*- *N*_16_	*N*_15_ - *N*_16_	*N*_15_ |*N*_16_
(*n* = 8)	⋮	⋮	⋮	⋮	⋮
**(B) Alone group**					
	**B-S**		**Learning**		**E-S**
	**(1-FS, 2-TS)**		**(2-FS, 18-TS, 2-WS)**		**(4-TS)**
(Alone)	*N*_21_		*N*_21_		*N*_21_
(*n* = 3)	⋮		⋮		⋮

*(A) B-S and E-S were performed alone whereas B-A, Learning and E-A were performed with a preassigned partner (another novice or an expert). (B) The novice participants always performed the task alone without dyadic interaction (FS, familiarization session; TS, training session; WS, wash-out session)*.

## 2. Methods

### 2.1. Participants

Twenty-three novice persons (12 male and 11 female, average age 26 ± 3.51 years) and an expert person (female, 33 years) participated in the study and provided written informed consent. Here, the novice participant has no previous knowledge about the task and the expert has been previously trained with the task under the external force field as an individual by performing 180 and 120 trials in two consecutive days. It is known that long practice with a task can result in expertise with the task (Magill and Anderson, 2010). In our study, the results demonstrated that performing 300 trials in total was enough to provide an appropriate level of expertise to a novice person. Two male and three female novice participants were left-handed, and the rest were right-handed according to the Edinburgh Handedness Inventory (Oldfield, 1971). All participants used their dominant hand in the study. The experiment was approved by the ethical committee of the University of Reading (No.SBS18-19 28). The experimental methods were performed in accordance with the relevant guidelines and regulations.

### 2.2. Experimental Groups

At the beginning of the experiment, all novice participants were randomly assigned to one of three experimental groups: Novice-Novice (*N-N*), Novice-Expert (*N-E*), and *Alone* (see Table 1). The *N-N* group consists of 12 novices. The participants in the *N-N* group were paired with another novice participant in the Learning phase. The *N-E* group consists of eight novices and they were paired with the expert in the Learning phase. The paired participants did not meet each other prior to or during the experimental sessions. In addition, 3 novices in the *Alone* group performed all trials as an individual. The primary study was the N-N vs. N-E, and the Alone group acts as a check-mark to ensure that no factors are inadvertently overlooked.

### 2.3. Experimental Setup and Task

To investigate “motor skill learning” and “adaptability to others,” in the light of the previous study (Mireles et al., 2017), we designed a cooperative visuo-haptic task as shown in Figure 1: a virtual mass (red circle) was connected to two cursors (blue and purple circles) by virtual springs (blue and purple lines). Visuo-haptic refers to the integration of visual information (e.g., the motion of two cursors on a screen) and haptic information (e.g., feeling the force arising from the virtual springs based on the cursor movement of the partner as shown in Figure 1).

The experimental setup includes two backdrivable haptic devices with two degrees of freedom to provide force feedback to participants to simulate the haptic tool for a given task. Through the haptic tool, they can interact with each other haptically. Encoders (HEDM-5500 Incremental Encoder) were attached to each joint to measure the position of the participant in the virtual environment and actuators (RE25 Maxon DC Motors) to generate forces to simulate the forces of virtual springs. Real-time control of the system was implemented with UDP Ethernet connection between Host PC and xPC Target by using MATLAB-Simulink software package (the Mathworks Inc., MA, USA).

The experiment was set up in two separate identical rooms equipped with a display, PC, and a haptic arm, and performed in two configurations (see Figure 1): single configuration to investigate skill learning and paired configuration for adaptability. As shown in Figure 1, in both configurations, they received the same visual feedback on the computer screen in which two joint cursors (blue and purple circles) and virtual mass (red circle) were presented. The motion of these two cursors was correlated with the motion of the end-effectors of the haptic interfaces as shown in Figure 1.

To assess the motor performance of the single novice participants in the *N-N* and *N-E* group (Figure 1), or to train oneself as a single in the *Alone* group (Figure 2), in the single configuration, the position of the cursor 2 was fixed at a certain location so that the single participants can control the cursor alone to bring the virtual mass to the target position. The novice participants were asked to perform a motor task as an individual by controlling cursor 1 which was correlated with a motion of the end-effector of the haptic arm. On the other hand, to assess the adaptability to others of the novice participants in the N-N and N-E groups, or to train the novice participants in the N-N and N-E groups, the novice participants were paired with a preassigned partner who sits in the next room.

In the study, the participants were asked to move a joint cursor (a 10 kg mass, visualized as a red circle on the screen) from a home position ([*x*_0_,*y*_0_]=[0,0]) to a randomly placed target as quickly as possible by controlling the robotic arm as a single or with their partner. Under this instruction, they must control the two cursors which are virtually connected to the virtual mass under an external unknown force field. The target appeared at one of eight locations equally spaced at 45 degrees on a circle with a radius of 60 around the home position of the joint cursor ([*x*_0_,*y*_0_] = [0,0]) in each trial. Successful target capture was adjusted as simply crossing the boundary into the target. The end-effectors of the robotic arms representing the blue and purple cursors were attached to the virtual mass *via* non-linear virtual springs generating two force vectors (${\vec{F}}_{c1}$ and ${\vec{F}}_{c2}$). To simulate the motion of a virtual mass with enough accuracy, the force of the virtual springs was calculated by considering two stiffness factors as (*k*_1_ = 148) and (*k*_2_ = 1480). *L*_1_ and *L*_2_ indicate the distance between the virtual mass and cursors (cursor 1 and cursor 2, respectively). Also, an external force field (${\vec{F}}_{ext}$) was applied to the virtual mass to simulate the unknown external force field for a motor learning task; the motion of the virtual mass in the virtual environment was affected by the force field. The stiffness factor (*k*_*ext*_) was set as 596 for the external force field.


(1) $$\begin{array}{c} {\vec{F}}_{ext}=k_{ext}\left[\begin{array}{cc} 1 & 0\\ 0 & -1 \end{array}\right]\left[\begin{array}{c} x_{m}-x_{0}\\ y_{m}-y_{0} \end{array}\right]\\ {\vec{F}}_{c1}=k_{1}L_{1}+k_{2}L_{1}^{2}\\ {\vec{F}}_{c2}=k_{1}L_{2}+k_{2}L_{2}^{2} \end{array}$$

These forces (${\vec{F}}_{ext}$, ${\vec{F}}_{c1}$, and ${\vec{F}}_{c2}$) were used to drive a mass (*M*)/damper (*B*) system (in 2 DoF).

(2) $$\begin{array}{r} M\frac{d^{2}{\vec{p}}_{m}}{dt^{2}}+B\frac{d{\vec{p}}_{m}}{dt}=\alpha {\vec{F}}_{ext}+{\vec{F}}_{c1}+{\vec{F}}_{c2} \end{array}$$

The experiment consists of five phases with three sessions: familiarization session (FS), training session (TS), and wash-out session (WS). The external force field was applied in the TS whereas the force was omitted during the FS and WS. Thus, α was used as a coefficient to activate or deactivate the external force field (${\vec{F}}_{ext}$) in the MATLAB program depending on the session. Namely, α was set as 1 in the TS whereas it was 0 in the FS and WS.

### 2.4. The Phases

The experimental protocol was set in five phases for the *N-N* and *N-E* groups (Figure 1) and three phases for the *Alone* group (Figure 2). All experimental phases consist of three sessions: (1) FS in which the participants were familiarized with the task without the effect of the external force field, (2) TS in which the participants were trained by performing the task under the external force field, and (3) WS in which the external force field was ignored to erase the learned motor skills (i.e., internal model of the partner). Each session consists of a number of target-set (TS) including eight trials, and each trial consists of a movement to bring a joint cursor from the home position to the target.

As shown in Table 1, the experimental paradigm includes five phases for the *N-N* and *N-E* groups (see Figure 1): The Baseline of the Skill Transfer (B-S) phase which indicates the individual baseline performance of each novice consists of one set of FS and two sets of TS. The Baseline of Adaptability (B-A) phase which can be considered as the baseline performance of the paired participants includes two sets of FS and one set of TS. As a next phase, the Learning phase including two sets of FS and 30 sets of TS was performed with another preassigned novice or with an expert to learn the cooperative task under the external force field. The novices in the *N-E* group performed the Learning phase with the expert whereas in the *N-N* group the participants paired with a new novice. For instance, as shown in Table 1, participant *N*_1_ executed the cooperative task with *N*_4_ instead of *N*_2_ in the Learning phase. Evaluation of Adaptability (E-A) phase which was performed as a pair to evaluate the individual adaptability of the novice participants includes two sets of FS, six sets of TS, and two sets of WS, respectively. The B-A and E-A phases were performed with the same pairs, namely if the B-A phase was executed by *N*_1_ and *N*_2_, the E-A phase was executed by the same participants e.g., *N*_1_ and *N*_2_. Last, Evaluation of Skill Transfer (E-S) phase including four sets of TS was performed as an individual.

For the *Alone* group (Figure 2), the experimental paradigm consists of three phases, and each phase was performed individually without the interaction with another one. B-S and E-S were performed in the same way as in the *N-N* and *N-E* group, but the learning phase was also performed as a single. The primary study was the comparison of *N-N* and *N-E* groups, and the *Alone* group was set as a check-mark to ensure that no factors are inadvertently overlooked. Also, by employing the *Alone* group, it is aimed to find the difference between trained with someone and learning through practice by oneself. The whole experiment was performed in 1 day; 3 h including 1 h break in the *N-N* group, 2 h including 45 min break in the *N-E* group, and 1 h with 15 min break in the *Alone* group. To prevent fatigue in the participants, there is a 15 s break between each target set (eight trials), 5 min break after each 10 target set, and, also, after each phase, there is a 30 min break.

To evaluate the motor learning to use the haptic tool under the unknown environmental dynamics and adaptability to a new novice participant, first, the B-S and E-S phases were compared to investigate how the novice participants can learn a new skill e.g., to practice by oneself, or with another novice, or with an expert. Second, the paired performances in the B-A and E-A phases were compared with the motor performance with a new novice in order to understand the adaptability to a new novice participant under the effect of corporation during the Learning phase.

### 2.5. Analysis and Statistics

To investigate the relationship between skill learning and adaptability within the motor learning paradigm, the performance of the participants was characterized by using three parameters (De Santis et al., 2014; Zenzeri et al., 2014; Mireles et al., 2017): (1) time to target (time duration to bring the virtual mass to the target position), (2) effort index (applied force to bring the virtual mass to the target), and (3) trajectory length (the length of the pathway followed by the participants to bring the virtual mass to the target). To quantify the motor learning and adaptability, using these parameters, two evaluation points were selected, namely, (1) the last set of TS in the baseline phase and (2) the first set of TS in the evaluation phase. To investigate the effect of training with a novice or an expert on motor learning, the two evaluation points were selected as the last target-set (TS) before the dyadic interaction and the first TS after the dyadic interaction. To evaluate the effect of the interacting partner during the Learning phase on adaptability to others, the evaluation points were selected as the last TS before the Learning phase and the first TS after the Learning phase. This means we compared the last TS of Baseline of Skill Transfer (B-S) phase and the first TS of Evaluation of Skill Transfer (E-S) phase to assess the skill learning and the last TS of Baseline of Adaptability (B-A) phase and the first TS of the Evaluation of Adaptability (E-A) phase for the adaptability. All analyses were performed by using SPSS and MATLAB software. A normality test (The Shapiro-Wilk test) was used to determine whether the samples were normally distributed (Royston, 1983) before the analysis. Wilcoxon Signed-Rank Test or a Paired-sample *t*-test was applied to evaluate the performance of the experimental groups. The significance level was set to 5%.

## 3. Results

**Figure 3:**
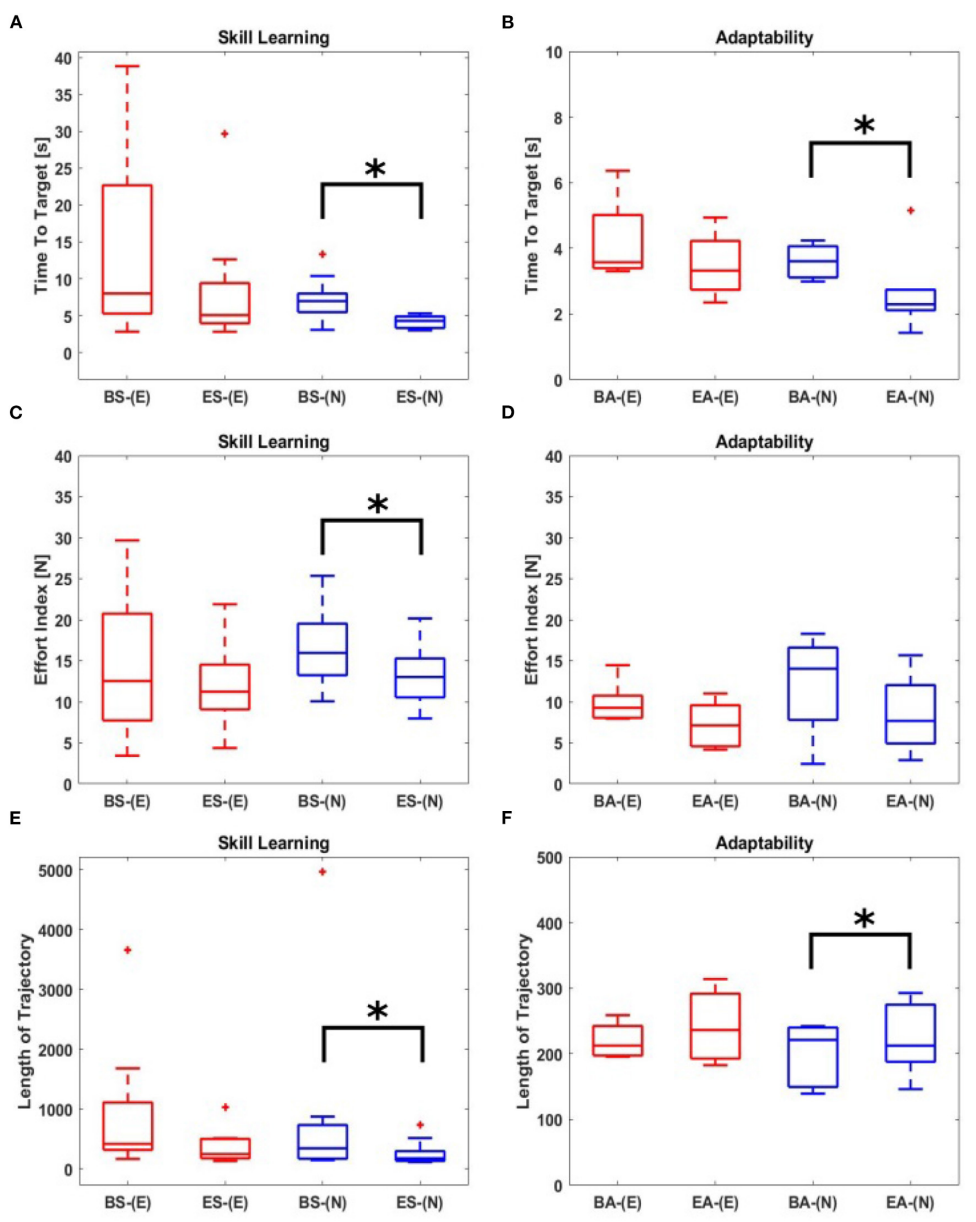
Summary of performance measures: experimental groups: *N-N* or *N-E*. Red box plots indicate the participant was paired with the expert. Blue box plots indicate the participant was paired with another novice. Asterisks denote significant differences (*p* < 0.05). “skill learning”; time points: the last TS of B-S [BS-(N) for *N-N* group and BS-(E) for *N-E* group] and the first TS of E-S (ES-(N) for *N-N* group) and ES-(E) for *N-E* group]; **(A)** “time to target” - N-N: *p* = 0.0020; N-E: *p* = 0.0781. **(C)** “effort index” - N-N: *p* = 0.0210; N-E: *p* = 0.6406. **(E)** “length of trajectory” - N-N: *p* = 0.0371; N-E: *p* = 0.0625. “adaptability”; time points: the last TS of B-A [BA-(N) for *N-N* group and BA-(E) for *N-E* group] and the first TS of E-A [EA-(N) for *N-N* group and EA-(E) for *N-E* group]; **(B)** “time to target” - N-N: *p* = 0.0332; N-E: *p* = 0.5576. **(D)** “Effort index” - N-N: *p* = 0.1943; N-E: *p* = 0.3279. **(F)** “Length of trajectory” - N-N: *p* = 0.0320; N-E: *p* = 0.1329.

As shown in Figure 1, in the *N-N* and *N-E* groups, the participants experiments consist of five phases in which the Baseline of Skill Transfer (B-S) and Evaluation of Skill Transfer (E-S) phases were performed sequentially to study the motor learning of the individual participants induced during the Learning phase. In the B-S and the E-S phases, the novice participants performed the task on their own without a partner to evaluate the motor learning. When comparing the last TS of the B-S phase and the first TS of the E-S phase, we found that the average of time to target (see Figure 3A) showed a significant decrease between the B-S and E-S phase in the *N-N* groups (*p* = 0.0020), however, not in the *N-E* groups (*p* = 0.0781). The decrease in time to target indicates the motor learning. This means that the novice in the *N-N* group has learned motor skill, i.e., how to control the haptic device under the unknown external field.

Also, it is important to note that, when changing the evaluation points in terms of skill learning, there is no difference in the results. For instance, when comparing the first TS of B-S and the first TS of E-S, there is a significant decrease in time to the target of the N-N group (*p* = 0.00098), however not in the N-E group (*p* = 0.3125). The result is the same when comparing the first TS of B-S and the last TS of E-S (N-N: *p* = 0.0244; N-E: *p* = 0.1484) or the last TS of B-S and the last TS of E-S (N-N: *p* = 0.0137; N-E: *p* = 0.1094).

When comparing the length of the trajectory (see Figure 3E), there is a significant difference in the *N-N* groups (*p* = 0.0371), however, no difference in the *N-E* (*p* = 0.0625) groups. When analyzing the effort index, the results corresponded to the previous results obtained by comparing the time to target and the length of the trajectory. It means that there is a significant decrease (*p* = 0.0210) in the effort index of the *N-N* groups (Figure 3C). Those results indicated that as the participants learned the task through the adaptation to the external force field, they explored the unknown force field to find the shortest pathway for a given task, which resulted in a decrease in the length of trajectory as well as a decrease in the time to target and effort index. This means that a novice participant can learn a new skill (to manipulate a tool under the unknown force field) with another person who has the same skill level through haptic interaction, i.e., mutual skill learning between two persons with the same skill level. However, on contrary to a common sense, skill transfer from an expert to a novice participant did not occur.

**Figure 4:**
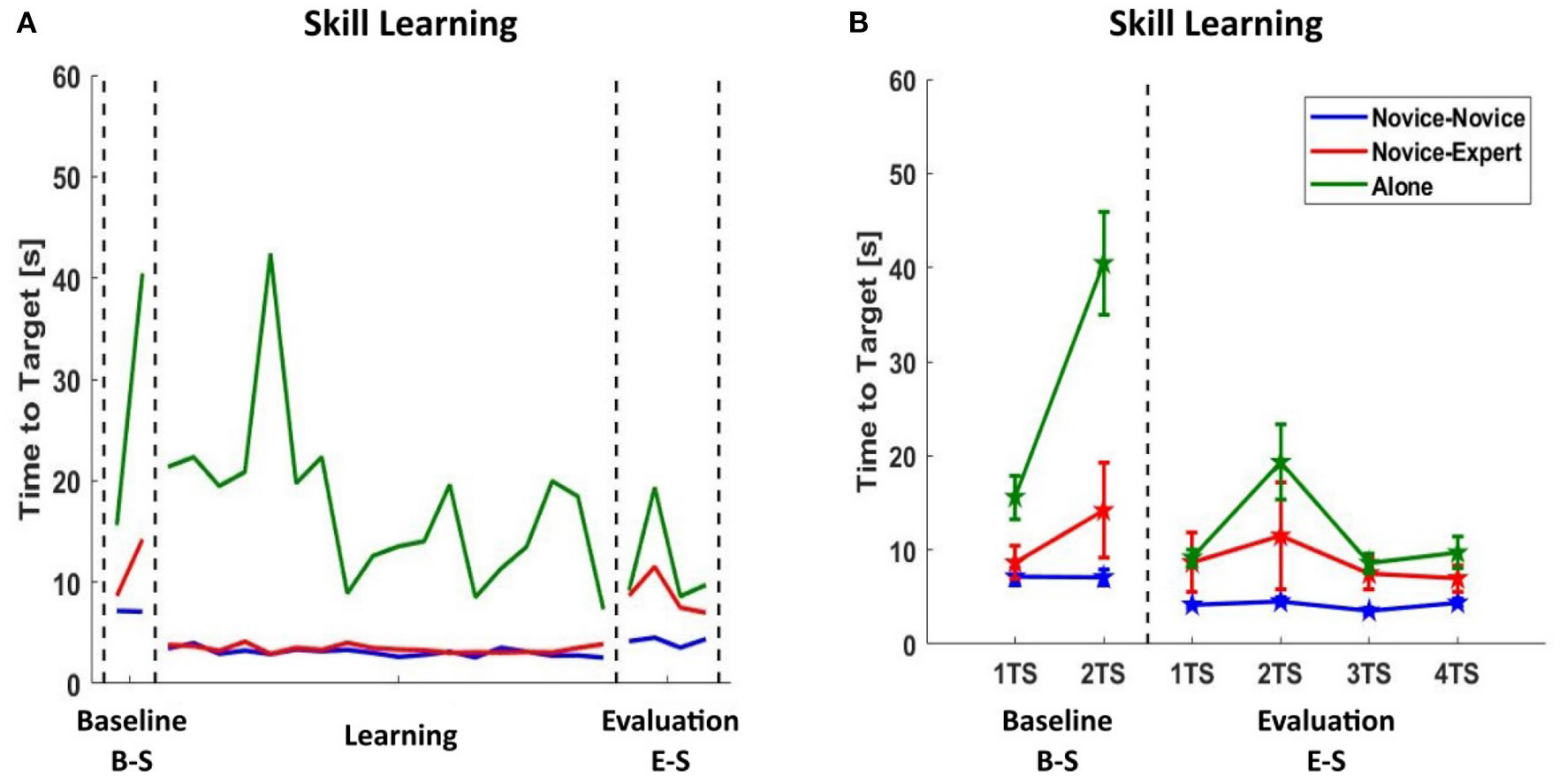
Comparison of an average of Time to Target in terms of skill learning in second (the duration to bring the virtual mass to the target position). *N-N* (blue), *N-E* (red), or *Alone* (green) - **(A)** Average variation of Time to Target between the first and the last target set in the three phases of the experimental protocol; baseline (B-S), learning and evaluation (E-S) phase in order. **(B)** Average of Time to Target in each target set (TS) of baseline (B-S) and evaluation (E-S) phase. The Baseline phase includes two TS, and the evaluation phase includes four TS. The dispersion bars represent the SE and are not shown on **(A)** to highlight the trend.

As a next step, we analyzed the dyadic performance of the *N-N* and *N-E* group by comparing the time to target, the effort index, and the length of trajectory in the Baseline of Adaptability (B-A) phase and the Evaluation of Adaptability (E-A) phase to evaluate the degree of the adaptability to a new partner. Here, the last TS before the Learning phase and the first TS after the Learning phase were used for evaluation. When applied a paired-sample *t*-test, the significant difference was seen in *N-N* groups (*p* = 0.0332), not seen in *N-E* groups (*p* = 0.5576). The average of time to target (Figure 3B) showed a decrease between the B-A and E-A phases in the *N-N* groups (B-A: 3.51±0.57; E-A: 2.17±0.48) as well a slight increase in the *N-E* groups (B-A:3.48±0.18; E-A: 3.86±0.95). The largest decrease in time to target (38.78 %) was found in the *N-N* group (Figure 4). In addition, when analyzing the length of trajectory in terms of adaptability (Figure 3F), the significant difference was seen in the *N-N* groups (*p* = 0.0320), not seen in the *N-E* groups (*p* = 0.1329). This result is consistent with the previous result (Figure 3B) obtained by comparing the time to target. In summary, the participants in the *N-N* group could induce better performance in adaptation to a new partner rather than those in the *N-E* group. That is to say, regarding skill learning and adaptability, the best improvement was found in the *N-N* group where the skill level was matched in the novice-to-novice interactions.

In order to understand whether the novice participants can learn the motor skill under the unknown environment on their own (learning as a single participant throughout), without the dyadic interactions (with another novice or the expert), the same motor task was also performed individually without the dyadic interaction in the Learning phase by fixing one of the cursors on the display. This group (practicing alone) serves as a reference group to make a comparison with other two groups with the dyadic interaction (N-N and N-E group). As shown in Figure 4, practicing in the *N-N* group (38.78% decrease in time to target) is the most advantageous option to learn motor skills. Also, practicing alone (37.75%) is better than practicing in the *N-E* group (19.16%).

## 4. Discussion

Human-human interaction in which your action affects the others, and the action of the others affects your action relies on continuous sensory feedback. The haptic sensory feedback being a channel for the mutual sharing of human intentions enables to achieve cooperative tasks.

We found that the best motor learning was induced in the N-N group, and the adaptability to others was best induced in the N-N group. This means that, according to our hypothesis, in peer-to-peer learning, adaptability to others could lead to the motor learning of the new tool under the unknown environment.

Previous studies (Ganesh et al., 2014; Mireles et al., 2017) using physical interaction based on haptic sensory feedback have shown that practicing with a partner is more advantageous than the individual practice to learn a task through unknown environmental dynamics. In our study, evaluating the performance as a single, the paired performance (learning the task together with the partner) showed better motor learning than the case of learning the task as a single participant. This result is consistent with the previous studies stating “two is better than one” (Ganesh et al., 2014; Mireles et al., 2017). However, in their study, two participants are not controlling the joint cursor, thus, not being aware of the mutual interactions. In addition, practicing with another beginner rather than an expert is better to learn a task and to improve the individual performance (Ganesh et al., 2014; Mireles et al., 2017). A recent study (Mireles et al., 2017) showed that the skill learning is possible through interaction with an expert in case of having prior experience with the task.

Imagine, you are asked to perform a motor task with your partner under unknown environmental dynamics. In such a case, there may be a dual instability to define the basis of the guiding force, e.g., partner or environmental dynamics. To date, the studies (Ganesh et al., 2014; Mireles et al., 2017) focused on the adaptation to the environmental dynamics, but “how a human adapts to a new partner in a cooperative task” still remains an unclear issue. In this study, we first investigated the effect of training with an expert or a novice partner on motor skill learning by utilizing the widely used paradigm (Mireles et al., 2017) and as a novel paradigm, we studied the ability to adapt to a new partner (adaptability). We hypothesized that if adaptability to others can be induced while attempting to achieve a common goal, motor learning under the unknown environment would occur simultaneously.

**Figure 5:**
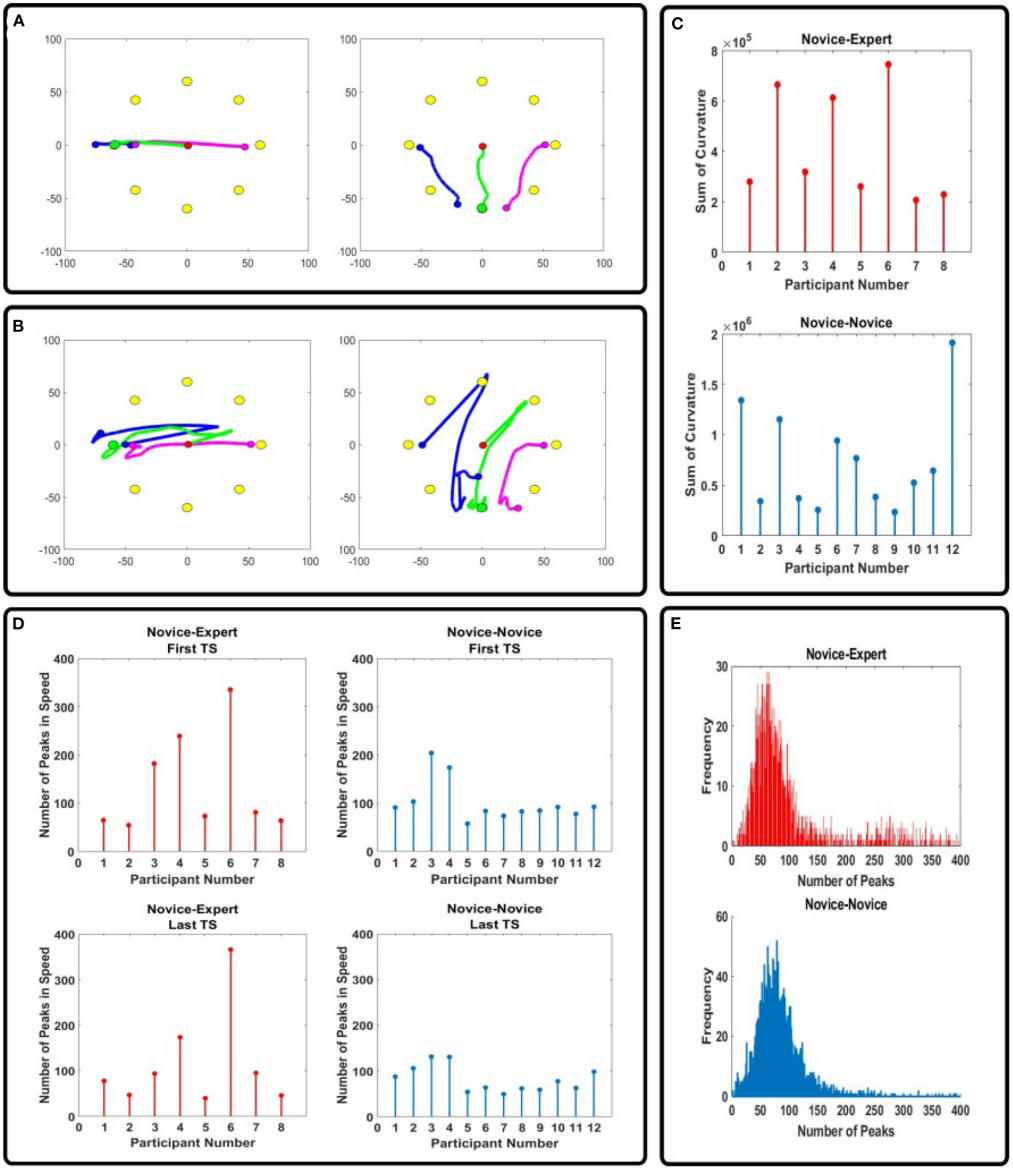
**(A)** Samples of the trajectories generated by *N-E* group in the Learning phase. **(B)** Samples of the trajectories generated by the *N-N* group in Learning phase. Green circle shows the final position of the virtual mass. After completing the task, the participants were asked to bring their cursors (blue and purple circles) to the starting point which was set as [−50,0] and [50,0]. The blue and purple line indicate the trajectories generated by the participants, and also the trajectories of the virtual mass is shown with the green line. **(C)** Sum of the curvature of the cursor trajectory during the Learning phase for each participant. **(D)** Change of the number of peaks in velocity between the first TS and last TS of the Learning phase. **(E)** Distribution of the number of peaks across all trials in the Learning phase.

**Figure 6:**
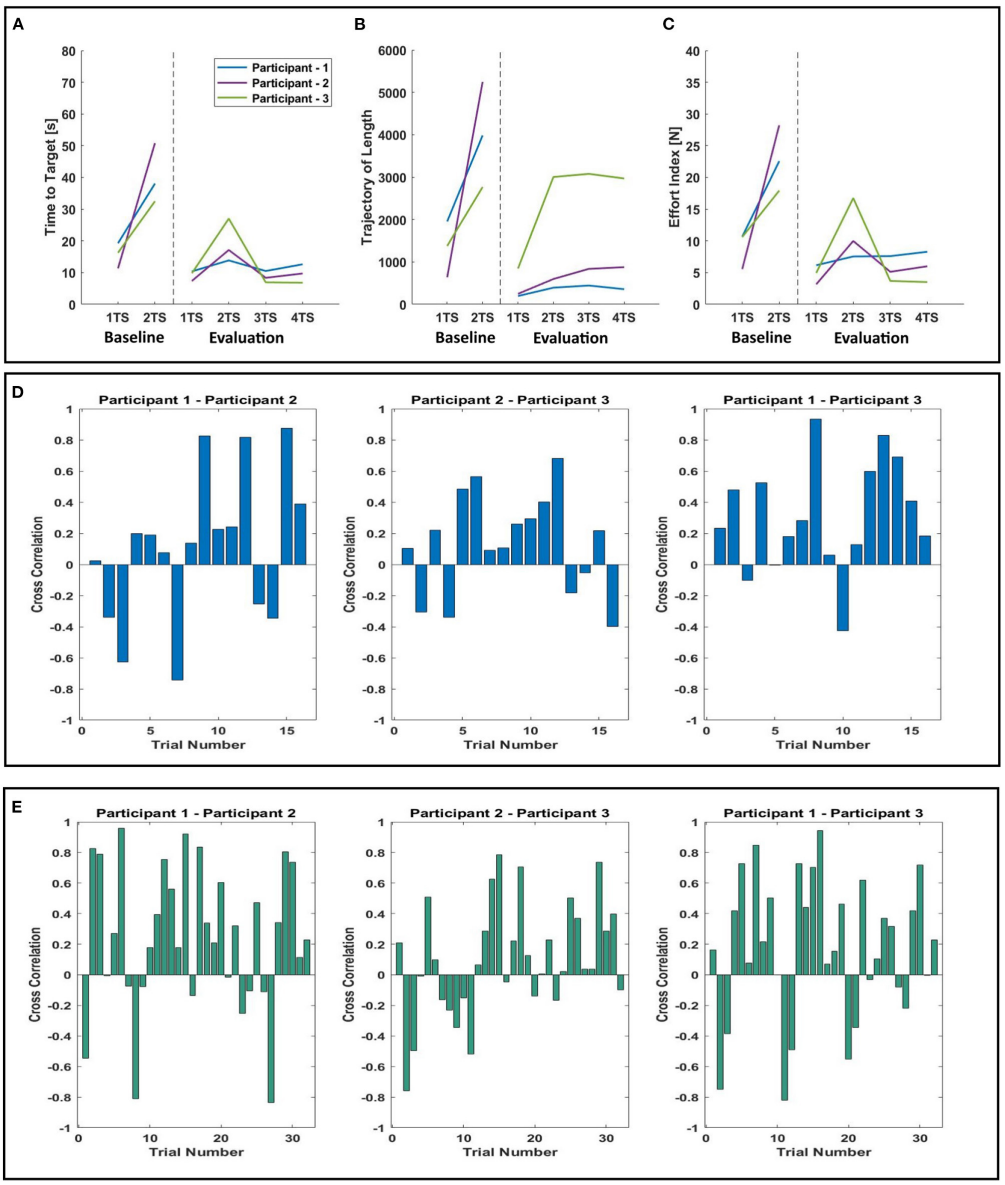
Summary of performance measures of *Alone* group. **(A)** Comparison of time to target, **(B)** comparison of trajectory length, **(C)** comparison of effort index, **(D)** cross correlation of trajectories in the Baseline (B-S) phase, **(E)** cross correlation of trajectories in the Evaluation (E-S) phase.

Our experimental results (Figure 3) demonstrated that practicing with another beginner during the Learning phase allows for skill learning through the mutual interaction with a partner. It is normally helpful to have expert guidance while learning a new skill, but we showed that the *N-N* group is better to learn skills when compared to the *N-E* group. Therefore, we next examined the reason for non-being skill transfer from the expert to the novice in our study. To this purpose, we analyzed the trajectories and the movement smoothness of the virtual mass controlled by the participants in the *N-N* and *N-E* groups in the Learning phase as shown in Figure 5. The results showed that the participants in the *N-N* group followed more complicated and longer trajectories (Figure 5B) than those in the *N-E* group (Figure 5A). When analyzing the sum of trajectory curvature (Figure 5C), which plays an important role in the analysis of point-to-point trajectories (Morasso and Ivaldi, 1982), the *N-N* group showed higher curvature (normal distribution with mean = 7.38 × 10^5^) than *N-E* group (mean = 4.14 × 10^5^). In addition, the number of peaks in speed (Figure 5D), which is one of measure of the movement smoothness (Rohrer et al., 2002), significantly decreased in the *N-N* group (*p* = 0.0048 <0.05). A decrease in the number of peaks in speed means an increase in movement smoothness, which shows, also, there is motor learning (Balasubramanian et al., 2015). When analyzing the distribution of the number of peaks across all trials in the Learning phase (Figure 5E), the *N-N* group has fewer peaks (a normal distribution with mean = 84.59) than the *N-E* group (mean = 121.20). In addition, when analyzing the trajectories followed by each participant in the Alone group, the correlation coefficient increased between the B-S and E-S phase, and the correlation is closer to 1 in the E-S phase when compared to B-S phase (Figure 6).

It can be summarized that the novice participants who are paired with another novice in the Learning phase could have more experience with the task by exploring the unknown external force field during the Learning phase. Thus, when doing the task alone in the evaluation phase, the participants could do the task more quickly, resulting in a significant decrease in time to target between the baseline and evaluation phase (Figure 3). Here, we can think of learning with another beginner as an example of “collaborative learning” (Dillenbourg, 1999), an educational approach in which two or more people make effort to learn the task together.

On the other hand, the novice participants interacting with the expert during the Learning phase could not have enough experience with the environmental dynamics which could be an obstacle to learning skills from the expert. Here, it is important to note that the expert must consider the point of view of the novice and ensure the feedback is in harmony with the novice's needs (Magill and Anderson, 2010). In conclusion, participants who performed the task with another novice can have more experience with the task due to the time for exploration (Figure 5), so they showed better results in skill learning.

In cooperative tasks, the other thing to be considered is predicting the motion of the partner at the next step. For such prediction, an internal model of the partner is necessary to simulate the motion of the partner in the action-perception loops. If one builds up the generalized internal model, one can adapt to a new partner and cooperate with them quickly to achieve a common task. Our results, being consistent with the hypothesis, indicate that novice-to-novice performance leads to mutual adaptability to others, which resulted in mutual skill learning. However, there is no skill transfer from an expert to a novice because of the lack of experience in the novice participants during the Learning phase, resulting in a lack of adaptability to a new partner. To summarize, more experience eases to explore the environmental dynamics and so find the internal model of the partner in the cooperative task. We speculate that the accumulation of the prediction errors defined as the difference between predicted and actual feedback can help in training individuals to develop the internal models of others. The prediction error allows a person to explore the “free energy” basin. Free-energy, which is a function of sensory and internal states, can be minimized through action (Friston, 2010). Minimizing free energy may increase the accuracy of predictions while performing the cooperative tasks.

In the activities requiring coordinated behaviors, it is important to explore the interpersonal synergies between the interacting people (e.g., adaptation between players and between a player and a conductor during ensemble music performance) to find the best way of skill learning. Our results are very informative for training session in the activities requiring cooperation and coordination such as dance (Chauvigné et al., 2019) or ensemble music performance (Wing et al., 2014). In modern society, though development of the industrial robots largely contributed to the manufacturing process of the products, for safety reasons, their work-space has been isolated from human operators. Thinking about the future of robots working in our daily environment, it is important to understand the nature of human-human interaction to create the human-machine interface which can be implemented in robots making a contact with humans for elderly care or rehabilitation (Sawers and Ting, 2014; Nasr et al., 2020). Thus, the principles of human-human interaction would facilitate the design of human-robot interfaces, “having ability to communicate naturally with humans as if humans do with each other” (Shimoda et al., 1999). That is to say, our findings will be useful to further investigate motor learning during human-human interaction (McNevin et al., 2000) and, also, to develop the human-machine interface which can be implemented in the control system (Sawers and Ting, 2014; Sakamoto and Kondo, 2015; Nishimura et al., 2021).

## 5. Conclusion

Our results showed that the experience with another novice partner during the Learning phase plays a significant role in adaptation to a new partner (adaptability) as well as a skill learning under an unknown field. That is to say, learning a motor task together with another novice through exploration of the unknown environmental dynamics led to higher adaptability for a person and to the best motor learning for a given task. We suggest that peer-to-peer learning would work, as they have more chances to explore the unknown external field, resulting in increasing the adaptability to others and learning the necessary skill set to control the device under the unknown field.

## Data Availability Statement

The raw data supporting the conclusions of this article will be made available by the authors, without undue reservation.

## Ethics Statement

The studies involving human participants were reviewed and approved by the Ethics Committees of the University of Reading (No. SBS18-19 28). The patients/participants provided their written informed consent to participate in this study.

## Author Contributions

TK and YH conceptualized the research project. OS did participant experiments, analyzed the data set, and discussed the results with WH, TK, and YH. All authors contributed to writing a draft of the paper and read and approved the final manuscript.

## Funding

This research was supported by Japan Society for the Promotion of Science Grants-in-Aid for Scientific Research (JSPS KAKENHI) (Grant numbers JP17KK0064, JP18K19732, JP19H05727, JP20H02111). This research project is also funded by the Ministry of National Education, the Republic of Turkey by means of a studentship.

## Conflict of Interest

The authors declare that the research was conducted in the absence of any commercial or financial relationships that could be construed as a potential conflict of interest.

## Publisher's Note

All claims expressed in this article are solely those of the authors and do not necessarily represent those of their affiliated organizations, or those of the publisher, the editors and the reviewers. Any product that may be evaluated in this article, or claim that may be made by its manufacturer, is not guaranteed or endorsed by the publisher.
